# A New Polyclinic

**Published:** 1910-11

**Authors:** 


### A New Polyclinic.

The New York Polyclinic Medical School and Hospital has recently filed plans for an eleven-story building to be erected on Fifteenth Street between Eighth and Ninth Avenues. The building will have a frontage of 95 feet and a depth of 96 feet.

The basement will contain a laundry, store rooms, and drug store. On the first floor will be a general reception hall for waiting patients, a reception room for private patients, a visitor's waiting room, a student's room, and offices for the trustees, the visiting staff, and the superintendent. On the second floor will be four wards, examining rooms, X-Ray rooms, laboratories, and a medical amphitheater. On the third floor will be four wards, rooms for the treatment of diseases of the eye, ear, nose and throat, minor surgery operating rooms, and the surgical amphitheater. On the fourth floor will be the maternity wards, the children's wards, and the museum. The fifth floor will contain rooms for the nurses and dormitories for the servants. On the sixth, seventh and eighth floors will be private rooms. The ninth floor will contain private operating rooms and wards. On the tenth floor will be isolation wards, servants, nurses and officers dining rooms. On the eleventh floor each class of convalescents will have its own solarium. The building is to cost $500,000, and will be constructed so as to allow for four additional stories.—*Exchange.*

---

A PASADENA woman recently placed her elegant residence in the hands of a real estate firm for rent. They rented it to a consumptive family, and one of this family died of tuberculosis in the house. The owner of the property then sued her agent for knowingly renting her house to a consumptive, asking $2200 damages. The judge awarded her $500. This decision will have an excellent effect. Agents have thus been taught a needed lesson.—*Southern California Practitioner.*

